# Motor Unit Fatigability following Chronic Carnosine Supplementation in Aged Rats

**DOI:** 10.3390/nu14030514

**Published:** 2022-01-25

**Authors:** Dawid Łochyński, Maciej Pawlak, Inge Everaert, Tomasz Podgórski, Magdalena Gartych, Anna-Maria Borucka, Jan Celichowski, Wim Derave, Dominik Kaczmarek

**Affiliations:** 1 Department of Neuromuscular Physiotherapy, Poznan University of Physical Education, 61-879 Poznan, Poland; lochynski@awf.poznan.pl; 2 Department of Neurobiology, Poznan University of Physical Education, 61-879 Poznan, Poland; gartych@awf.poznan.pl (M.G.); celichowski@awf.poznan.pl (J.C.); 3 Department of Physiology and Biochemistry, Poznan University of Physical Education, 61-879 Poznan, Poland; pawlak@awf.poznan.pl (M.P.); podgorski@awf.poznan.pl (T.P.); am.borucka@gmail.com (A.-M.B.); 4 Department of Movement and Sports Sciences, Ghent University, 9000 Ghent, Belgium; Inge.Everaert@UGent.be (I.E.); Wim.Derave@UGent.be (W.D.)

**Keywords:** carnosine, aging, motor units, fatigue, rat

## Abstract

Studies suggest that carnosine (beta-alanyl-L-histidine) is effective in treating neuromuscular diseases associated with aging, but there is still a need to clarify its role in motor units (MUs) function during aging. In this study, 40 male Wistar rats aged 15 months were randomly assigned to a control or to two experimental groups in which 0.1% carnosine supplementation was performed for 10 or 34 weeks. After 34 weeks, we examined fast fatigable (FF), fast fatigue-resistant (FR) and slow (S) MUs’ force properties and fatigability, as well as antioxidant potential, advanced glycation end products, activity of enzymes, and histidyl dipeptides content in the medial gastrocnemius muscle. Short- and long-term carnosine supplementation maintained the force of FF MUs at a higher level during its rapid decline seen from the initial 10 to 70 s of the fatigue test. In FF, especially long-term, and in FR MUs, especially short-term, carnosine supplementation resulted in less rapid force decline during the initial 70 s of the second fatigue protocol. Carnosine supplementation did not change muscle antioxidant potential and mortality rate (~35% in all groups), nor muscle mass with aging. Moreover, instead of the expected increase, a decrease in histidyl dipeptides by ~30% in the red portion of medial gastrocnemius muscle after long-term supplementation was found. After chronic carnosine supplementation, the specific changes in fatigue resistance were observed in FF and FR units, but not in S MU types that were not accompanied by an improvement of antioxidant potential and activity of glycolytic or oxidative enzymes in aged rats. These observations indicate that carnosine supplementation during aging may generate different physiological adaptations which should be considered as an important factor when planning treatment strategies.

## 1. Introduction

Aging is associated with an excessive production of reactive oxygen and/or nitrogen species in skeletal muscle [1,2] which leads to overactivation of specific redox-sensitive signaling pathways involved in sarcopenia, and results in muscle weakness and decreased functionality [3,4]. Aging also contributes to the accumulation of advanced glycation end products (AGEs) in both intracellular and sarcolemmal muscle cell domains [5], a process which likely contributes to an age-related muscle degeneration and decline in function [6].

A decrease in glycolytic phosphorylation enzymes such as hexokinase and lactate dehydrogenase activities, as well as an increase in pyruvate kinase, was previously noted in distinct human skeletal muscles with aging [7]. The first one is a very important determinant of skeletal muscle ability to sustain contractile activity with a moderate intensity, and reduction of this enzyme impairs endurance capacity [8]. Similarly, lower activity of oxidative enzyme citrate synthase has been noted in specific older lower limb muscles (i.e., gastrocnemius), despite previously reported shifts of the muscles towards a more oxidative profile with the aging process [9]. This suggests that, compared to the young, older individuals may have lower fatigue resistance to short- and long-lasting activity in isolated motor units, keeping a similar glycolytic or oxidative muscle fiber profile.

A number of previously published studies have indicated that some specific muscles of old men fatigue relatively less than those of younger men during both isometric and dynamic volitional contractions [10]. However, other results have indicated no difference [11], or even an increase in fatigability [12]. Studies carried out in rodent muscle [13] or isolated motor units, composed of predominantly uniform muscle fiber types [14], have shown unaltered or decreased fatigue resistance during moderate isolated contractile activity evoked by electrical stimulation. 

Mammalian skeletal muscles contain a relatively high millimolar concentration of two histidyl dipeptides (beta-alanyl-L-histidine), carnosine and its methylated analogue, anserine. Concentration of these histidyl dipeptides is higher in fast-twitch than in slow-twitch skeletal muscle fibers [15], but their role in contractile function is important for both fast [16] and slow fibers [17,18]. In the 1960s, carnosine had been considered to have anti-aging abilities [15], but to date there are only a few studies that have actually evaluated this hypothesis. McFarland and Holliday [19] showed that carnosine can prevent the onset of senescent cell morphology and rejuvenate senescent cells in a human diploid fibroblast in vitro. Carnosine was also supposed to improve the long-term survival of Escherichia coli through its antiglycation effects [20]. Similarly, consumption of 200 mg/L or even 50 mg/L carnosine in standard foodstuffs increased the life span of the male fruit fly, Drosophila melanogaster, by 20% [21,22]. Another study of Yuneva et al. [23] showed that carnosine can also increase the life span and improve biochemical indices related to aging in mammals, as the 50% survival rate was increased by 20% and associated with the decrease in activity levels of Na/K-ATPase, monoamine oxidase b, and tiobarbituric acid reactive substances (TBARS). In aged rats, so far one study has shown that a one-month intraperitoneal treatment with 250 mg/kg carnosine results in decreased serum LDL, VLDL, erythrocyte malondialdehyde, and diene conjugate levels. This indicates that carnosine treatment has the potential to lower aged-induced oxidative stress [24]. However, there is a need to investigate if supplementation of carnosine is effective in managing sarcopenia during aging, which involves the loss of muscle mass and function [25].

In the last two decades there has been growing evidence that muscle carnosine loading through beta-alanine supplementation improves muscle performance during high-intensity activities in sportsmen [26]. Kaczmarek et al. [18] have shown that in young adult rats, the muscle carnosine and anserine loading induced by beta-alanine results in an increase in twitch amplitude of fast fatigable (FF) units and maximum tetanic force of fast resistant (FR) units in medial gastrocnemius muscle. There was also a lower decline in force of slow (S) motor units evoked by repeated low-frequency stimulation. Furthermore, beta-alanine supplementation increased the gastrocnemius muscle carnosine content in healthy elderly subjects and caused an increased time-to-exhaustion during the constant-load submaximal or incremental physical capacity tests [27].

Based on the previous studies, we formulated the following research questions: (1) can carnosine supplementation result in lesser fatigability and reduced low-frequency fatigue of slow MUs in aged rats; (2) can carnosine supplementation have systemic effects and increase muscle antioxidant or anti-glycating potential, as well as increase survival rate with aging; and (3) does the duration of carnosine supplementation differently affect these potential effects? In this study, using a well-known animal model and objective electrophysiological and biochemical methods, we aimed to assess short- or long-lasting carnosine treatment in sedentary rats. For this purpose, aged rats were orally supplemented with carnosine for 10 weeks and 34 weeks. Our hypothesis was that carnosine supplementation would increase antioxidant potential and muscular metabolic capacity in rat skeletal muscle and this in turn would result in lesser fatigability, especially of fast MUs, and be more pronounced after long-term supplementation. As carnosine loading decreased the low-frequency fatigue of slow MUs in young animals, we supposed that this effect would also be present in old rats after carnosine supplementation. Carnosine is known to have both ergogenic and systemic effects; therefore, we assumed that carnosine supplementation increases muscle antioxidant anti-glycating potential and survival rate with aging.

## 2. Materials and Methods

### 2.1. Ethical Approval

The animal care and treatment procedures followed the Guiding Principles for the Care and Use of Animals in the Field of Physiological Sciences, the European Union guidelines, and the Polish Law on the Protection of Animals. Local Ethics Committee in Poznan at the Poznan University of Life Sciences, Poland approved (No. 67/2015) the experimental procedures.

### 2.2. Animals

The study was performed on 40 male Wistar rats (Rj:WI, SPF Han, Janvier Labs, outbred stock) purchased at 15 months of age. Their initial body weight was 663 ± 63 (SD) g. The animals were housed two per cage. The light-dark cycle (12:12-h), temperature (22 ± 2°), and humidity (55 ± 10%) of the room were controlled. Throughout the study period, the animals had unrestricted access to water. They were fed with commercial rat chow which did not contain carnosine or derivatives. This ensured a standard nutrient diet.

### 2.3. Study Design

After 2 weeks of acclimatization, animals were randomly assigned to either a control, non-supplemented group (CON, *n* = 14), or to two experimental groups in which animals were treated with carnosine for either 34 weeks (CAR34W; *n* = 14) or for the last 10 weeks of the study (CAR10W; *n* = 12). Two weeks after the adaptation period, rats in the first experimental group started an 8-month period of supplementation with 0.1% wt/vol carnosine (Flamma S.p.A., Chignolo D’isola, Italy) solution in their drinking water (1 g/L, which in our study corresponded to ~46.3 mg/kg body wt/day). In the second experimental group, short-term supplementation with 0.1% carnosine (~46.0 mg/kg body wt/day) began 10 weeks before the end of the study in order to ensure the same age of animals in all groups during the period of the electrophysiological experiments. Two times per week, i.e., on Tuesday and Friday, bottles were cleaned and replaced in all cages and fresh water, with or without the addition of carnosine, was prepared to ensure safety and hygiene. Further, each week, the body mass of all animals and food and water intake were registered. After 34 weeks of study, in all animals that survived, muscle samples were collected and 6, 8, and 8 rats were used in electrophysiological experiments in the CON, CAR10W, and CAR34W groups, respectively. 

### 2.4. Surgical and Electrophysiological Procedures

Anesthesia was induced by administration of sodium pentobarbital (Morbital, initial dose 60 mg/kg) intraperitoneally. It was maintained throughout the experiments by additional doses of 10 mg/kg, and supplemented approximately every hour. The strength of pinna and limb withdrawal reflexes was continuously monitored to control its depth [28]. At the end of the experiments, a lethal dose of pentobarbital (180 mg/kg) was administered. At the beginning of the experiment, the left medial gastrocnemius was carefully dissected and freed from its surrounding tissues in order to preserve the innervation and vessels. Next, the laminectomy was performed and L4 and L5 ventral roots were cut and exposed [28]. The muscle was connected to an inductive force transducer with a non-elastic thread. To obtain optimal contractile conditions in the majority of MUs, the muscle was stretched up to a passive force of 100 mN [29]. MU contractile force was tested in isometric conditions. The splitting of ventral roots into thin filaments was performed to separate axons of alpha motoneurons. The filaments were stimulated (Dual Output Square Pulse Stimulator, model S88; Grass Instrument) with square electrical pulses (0.1-ms duration) of variable voltage (up to 0.5 V). During the process of isolation of single MUs, the strength of the electrical stimulation was adjusted until the “all-or-none” type action potential was recorded with bipolar silver wire electrodes inserted into the muscle and the MU twitch force had the same shape and size [18]. The threshold of electrical stimulation and passive muscle preloading were monitored during MU stimulation [18]. A closed-loop temperature controller was used to automatically maintain animal body core temperature at a constant level of 37 ± 1 °C in order to ascertain the same conditions of MU activation throughout the time of the electrophysiological experiments.

### 2.5. Testing Protocol and Data Analysis

At the beginning of the stimulation protocol, five twitches were evoked with one second time intervals. Then, one unfused tetanus contraction (500 ms, 40 Hz) and one maximum tetanus contraction (300 ms, 150 Hz) were administered. MUs with a sag in the force of the unfused tetanus contraction evoked at 40 Hz were classified as fast MUs. Those without the sag were classified as slow MUs [30,31]. In both fast and slow MUs, these three successive steps of stimulation were separated by 10-s breaks and followed by the first (standardized) 4-min fatigue test protocol. It was composed of 40 Hz stimulation trains, which lasted 325 ms, and were repeated once per second [18]. In fast MUs, the same, 3-min fatigue protocol was applied after a 3-min break. In slow units, a 4-min, low-frequency protocol was administered after a 30-s break. It consisted of 20-Hz frequency trains, which lasted 350 ms, and were repeated once per second [18]. After the second fatigue test, the post-fatigue recovery protocol was implemented. It was composed of seven 40 Hz trains of stimuli lasting 325 ms for fast MUs and seven 20 Hz trains lasting 350 ms for slow units. The trains were repeated at 10-s intervals. There was a 1-s break between the second fatigue test and recovery protocol [18]. From the averaged twitch response, the peak twitch force, time of contraction, and time of half-relaxation were measured for each MU. To calculate the twitch-to-tetanus force ratio, the maximum tetanus force was also measured. The reverse ratio of the initial peak tetanic force to the peak force generated 2 min later was calculated during the first fatigue test as a measure of the fatigue index [18]. Fast motor units with an index score of below 0.5 were classified as FF and units with an index score exceeding 0.5 were classified as FR [31,32].

To express the fatigability of all types of MUs, the peak forces of 40 Hz tetanic contractions were measured every 10 s, both during the first and the second fatigue tests. For both tests, these forces were expressed as a percentage of peak force of the second tetanus evoked during the first fatigue test. In slow units, in the first and the second fatigue tests, the peak tetanic forces measured were expressed in relation to the peak force of the first tetanus in each test. Additionally, force measurements in the first fatigue test were performed every second during the initial 10 s for fast and initial 20 s for slow MUs to obtain a more detailed analysis of fatigability [18]. The course of the fatigue test of fast units was divided into three theoretical time zones of energy production by ATP regeneration: (1) from 0 to 10 s, when the phosphagen metabolism dominates; (2) from 10 to 70 s, when glycolysis prevails; and (3) from 70 s to the end of the tests, when mitochondrial respiration predominates during contractile activity [33].

### 2.6. Antioxidant Potential, Advanced Glycation End Products, and Enzyme Activity in MG Muscle

Immediately after the electrophysiological experiments, the left medial gastrocnemius was removed, weighed, placed into cryogenic vials (NUNC/Thermo Fisher Scientific, Roskilde, Denmark), frozen in liquid nitrogen, and stored at −80 °C until analysis. Subsequently, homogenization was performed by dissolving the collected muscle samples in phosphate-buffered saline (PBS) at a 1:9 tissue to buffer ratio. The samples were homogenized with 28,000 to 30,000 rpm using VWR VDI 12 disperser homogenizer (Singapore). After centrifugation, the removed supernatant was stored in Eppendorf tubes at 4 °C until assayed.

In order to determine the possible antioxidant potential of carnosine, the ferric reducing ability of plasma (FRAP), total phenol substances (TP), and thiobarbituric acid reactive substances (TBARS) were measured in the left medial gastrocnemius muscle. FRAP concentration was measured using a method initially developed by Benzie and Strain [34] and modified by Janaszewska and Bartosz [35]. Absorbance was measured using a multi-mode microplate reader (Synergy 2 SIAFRT, BioTek Instruments, Winooski, VT, USA), λ = 593 nm, and concentration was expressed as µmol/L of muscle supernatant. TP concentration was assessed with the method described by Singleton and Rossi [36], using the ability of the Folin-Ciocalteau reagent to oxidize phenol groups, and detected with a multi-mode microplate reader (Synergy 2 SIAFRT, BioTek Instruments, Winooski, VT, USA) at 765 nm. TP concentration was expressed as an equivalent of gallic acid in g/L of supernatant. Finally, TBARS concentration was assessed according to Ohkawa et al. [37], detected with a multi-mode microplate reader (Synergy 2 SIAFRT, BioTek Instruments, Winooski, VT, USA) at λ = 532 nm, and expressed as µmol/L of supernatant.

To assess the anti-glycation potential of carnosine, advanced glycation end products (AGEs) content was determined in the left medial gastrocnemius with the use of a commercially available fluorescence kit (Advanced Glycation End Products (AGEs) Assay Kit, BioVision, CA, USA, catalogue no. K929-100). In addition, the activity of the main enzymes involved in muscle contraction biochemistry, i.e., hexokinase (Hexokinase Colorimetric Assay kit, BioVision, CA, USA, catalogue no. K789-100), kinase pyruvate (Pyruvate Kinase Activity Colorimetric Assay Kit, BioVision, CA, USA, catalogue no. K709-100), and citrate synthase (Citrate Synthase Activity Colometric Assay Kit, BioVision, CA, USA, catalogue no. K318-100) was assessed. A multi-mode microplate reader (Synergy 2 SIAFRT, BioTek Instruments, Winooski, VT, USA) was used to measure fluorescence at Ex/Em = 360/460 nm for AGEs, at 450 nm for hexokinase, at 570 nm for pyruvate kinase, and at 412 nm for citrate synthase. For calculation of the AGE amount in the supernatant, the fluorescence (Ex/Em = 360/460 nm) of the background control (1 mg/mL BSA) was used as a reference, defined as 1 arbitrary unit (AU) and expressed as AU/mg of supernatant protein. Sample hexokinase and pyruvate kinase activity was expressed as mU/mL, while in the case of citrate synthase, the activity was expressed as U/mL.

### 2.7. Muscle Content of Carnosine, Anserine, and Taurine

Each experiment started with the careful dissection of the right medial gastrocnemius muscle. Connective or fat tissues were carefully removed. The medial gastrocnemius muscle was divided into the red part, predominantly containing type I muscle fibers, and into the white part, mainly containing type II muscle fibers. The acquired muscle samples were weighed and put into cryogenic vials (NUNC/Thermo Fisher Scientific). They were immersed in liquid nitrogen and frozen at once. Until analysis, they were kept at −80 °C. Subsequently, homogenization was performed by dissolving the muscle samples in phosphate buffer saline solution (PBS, 20 µL/mg muscle) [18]. Deproteinization of muscle homogenates was done using 35% sulfosalicylic acid and centrifugation with 16,000× *g* for 5 min. Then, the 5 KL of deproteinized supernatant and 75 KL AccQ Fluor Borate buffer were mixed together. The combined standard solutions of carnosine, anserine, and taurine (Sigma) were treated in the same way. The following parameters of a Waters high-performance liquid chromatography system were applied to the derivatized samples: XBridge BEH column (4.6 × 150 mm, 2.5 Km) for the quantification of carnosine, anserine, and taurine, with fluorescence detector. The excitation/emission wavelength was 250/395 nm. The equilibration of columns was performed at room temperature with the buffer A (10% eluent A (Waters), 90% H_2_O), buffer B (100% acetonitrile), and buffer C (100% H_2_O) at a flow rate of 1 mL/min [38].

### 2.8. Statistical Analyses

Data are presented as means ± SD, and *p* < 0.05 was considered statistically significant. Data normality was assessed with the Shapiro–Wilk test. In order to verify the proportions of isolated MUs in each studied group, the populations of sampled MUs were assigned to three MU categories, which were compared with the use of the Pearson’s chi-square test. The Mann–Whitney U test was used to compare carnosine intake between the two experimental groups.

Comparisons of mean values between the three groups were made with the use of one-way measures analysis of variance (ANOVA) or Kruskal–Wallis tests for the following data: MG muscle weight, twitch force, contraction time, half-relaxation time, maximum tetanus, and muscle concentration of carnosine, anserine, taurine, FRAP, TP, TBARS, and AGEs, as well as for the activity of hexokinase, kinase pyruvate, and citrate synthase. In order to check the ANOVA assumptions, Levene’s test for equality of variances was used. When ANOVA was significant, multiple post hoc tests, after the Bonferroni correction, were performed. For data where normality of data was not met or the variances were unequal, the Kruskal–Wallis test, followed by Dunn’s multiple comparison test, was applied.

As the ANOVA assumptions were met, the differences in the values of body weight and food and water intake, as well as in the force profiles during both fatigue tests and recovery period, were tested with the two-way repeated-measures ANOVA with time (0–35 weeks for weight and food and water and stimulation times for fatigue and recovery protocols), group (CAR10W vs. CAR34W vs. CON), and the interaction between the time and group as factors. The homogeneities of variance were assessed using the Mauchly’s sphericity method. The Greenhouse–Geisser correction was used when the assumption on data sphericity was violated. Post hoc multiple comparisons were made with the Bonferroni-adjusted *t*-test. 

## 3. Results

### 3.1. MUs Number and Contractile Properties

**Figure 1:**
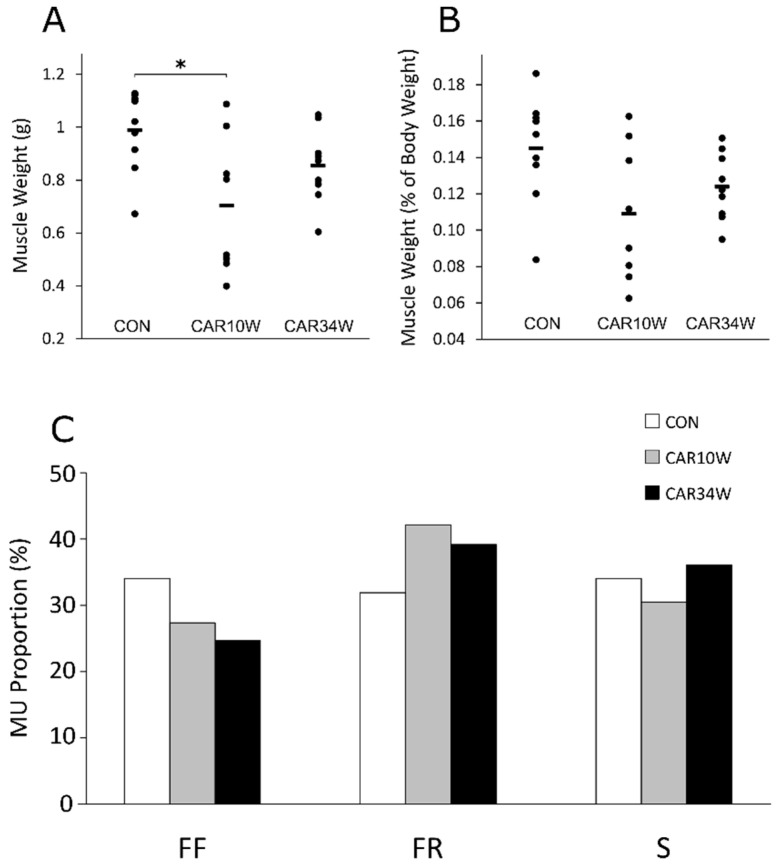
Medial gastrocnemius absolute and normalized muscle weights and distribution of motor units in CON, CAR10W, and CAR34W rats. (**A**) Absolute muscle weight (g). (**B**) Muscle weight normalized to body weight (%). (**C**) Percentage of different types of MUs. Mean ± SD values are presented in (**A**,**B**). The asterisks above the bars denote a significant difference (*p* < 0.05; one-way ANOVA Bonferroni-corrected post hoc test). CON refers to the non-supplemented group, CAR10W to the 10 weeks supplemented group, and CAR34W to the 34 weeks supplemented group. Mus: motor units.

Very similar numbers of motor units were recorded and analyzed in each group, i.e., 94, 95, and 97 in the CON, CAR10W, and CAR34W groups, respectively. In all studied groups, a comparable proportion of FF, FR, and S units (40 vs. 26 vs. 29 in CAR10W, 38 vs. 24 vs. 35 in CAR34W, and 30 vs. 32 vs. 32 MUs in the CON group, respectively) were collected (Pearson’s chi-square test: 3.352, df = 4, *p* = 0.501, see Figure 1C). No alterations in the studied contractile time and force-related parameters were noticed, except that the twitch force was significantly increased in the slow MUs of the CAR34W rats with respect to the CON rats (Table 1).

### 3.2. MUs Fatigability

#### 3.2.1. FF Units

**Figure 2:**
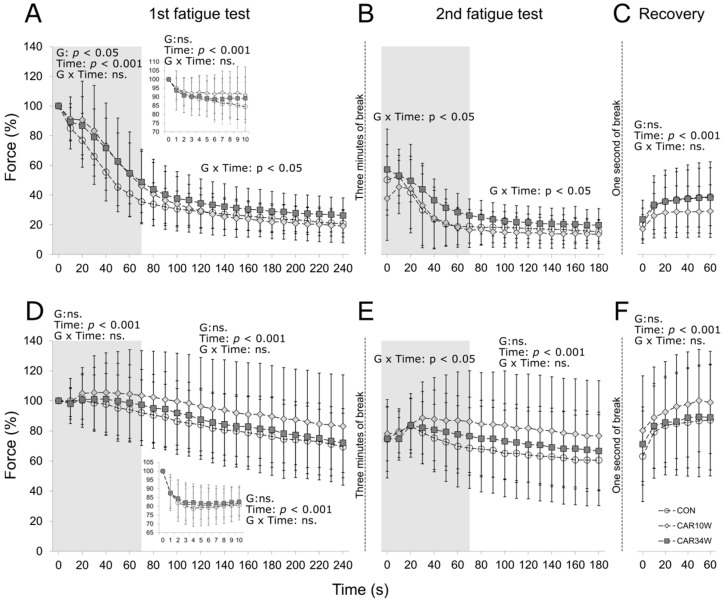
Effects of carnosine supplementation on the mean peak relative forces of unfused 40-Hz tetani during applied stimulation of FF and FR MUs. Peak forces were presented every 10 s and expressed as percentage of the peak force of the first tetanus (100%) generated at the beginning of the first fatigue test. The inset panels in A and D present forces generated every second during the first 10 s of stimulation. Panels (**A**–**C**) represent the force profiles of FF MUs and panels (**D**–**F**) represent the force profiles of FR MUs during the first and second fatigue tests and the recovery periods. Theoretically, the grey zone represents the dominant source of energy production from glycolysis and the white zone that from mitochondrial respiration, while the inset panels represent that from phosphagen systems (see Section 2 for explanation). CON refers to the non-supplemented group, CAR10W to the 10 weeks-supplemented group, and CAR34W to the 34 weeks-supplemented group.

In the initial 10 s of the first fatigue protocol, there was no effect of group (F(2,693) = 2.500, *p* = 0.088; inset panel in Figure 2A), nor of group times time interaction (F(18,693) = 1.392, *p* = 0.238; inset panel in Figure 2A) on force profile. Between 10 and 70 s of the first fatigue test (when glycolysis is the main source of ATP regeneration), force was maintained at the higher level in the supplemented animals (statistically significant effect of group, F(2,450) = 4.877, *p* = 0.010), but there was no time times group interaction (F(12,450) = 1.541, *p* = 0.190) (grey zone in Figure 2A). From the 70th until the 240th s of the test, when mitochondrial respiration begins to be a dominant source of energy production, force declined more in the supplemented groups (especially in CAR10W) (significant group times time interaction F(34,1275) = 3.664, *p* = 0.015). The effect of group (F(2,1275) = 1.864, *p* = 0.162) on force generation was not significant (white zone in Figure 2A).

During the initial 70 s of the second fatigue test, force declined at lower rates (especially in CAR34W) (significant effect of group × time interaction F(14,497) = 2.425, *p* = 0.026), but was not maintained at higher levels (no effect of group F(2,497) = 2.407, *p* = 0.097) in the supplemented groups as compared to the control animals (grey zone, Figure 2B). The opposite trend was observed between 70 and 180 s, as force declined more in CAR34W than in the other groups (group × time interaction on force profile was significant (F(22,781) = 2.321, *p* = 0.035), but without the effect of group (F(2,781) = 2.707, *p* = 0.074)).

Finally, there were no significant group × time interaction (F(12,426) = 1.481, *p* = 0.228, Figure 2C) nor group (F(2,426) = 1.656, *p* = 0.198, Figure 2C) effects on force levels during the recovery trains applied at the end of the fatigue protocol.

#### 3.2.2. FR Units

No effects on force developed were found when the first 10 s of the fatigue test were analyzed (effect of group, F(2,882) = 0.955, *p* = 0.388; group × time interaction, F(18,882) = 0.162, *p* = 0.965; inset panel in Figure 2D). Between 10 and 70 s of the first fatigue test, relatively comparable and stable forces of FR units for all groups were noted and no effect of group (F(2,570) = 0.994, *p* = 0.374) nor group × time interaction (F(12,570) = 2.068, *p* = 0.120; grey zone in Figure 2D) were found. In the third phase of the fatigue test (70–240 s), a 10–15% decrease in force was observed, on average, in all groups, but also no effect of group (F(2,1615) = 2.053, *p* = 0.134) nor group × time interaction (F(34,1615) = 0.475, *p* = 0.723; white zone in Figure 2D) were noted.

During the first 70 s of the second fatigue test, the forces of FR units of control animals declined more than in supplemented animals (time × group interaction, F(14,637) = 2.654, *p* = 0.048; grey zone in Figure 2E). The effect of group (F(2,637) = 1.646, *p* = 0.198) on force generation was not significant. Between the 70th and 180th second, the force of FR units declined, with the same rate in all groups, and no group × time interaction (F(22,1001) = 0.461, *p* = 0.878; white zone in Figure 2E) nor group effect (F(2,1001) = 2.493, *p* = 0.088) were found. Finally, there was no group effect (F(2,528) = 1.144, *p* = 0.323) nor group × times interaction (F(12,528) = 1.979, *p* = 0.093; Figure 2F) during the recovery trains applied at the end of the fatigue protocol.

#### 3.2.3. Slow Units

**Figure 3:**
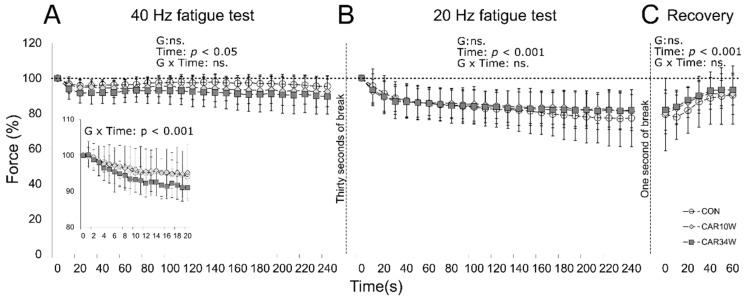
Effects of carnosine supplementation in the course of the first (**A**) and second (**B**) fatigue tests, as well as the recovery period (**C**), in slow MUs. Peak forces were presented every 10 s and expressed as percentages of the peak force of the first tetanus (100%) generated at the beginning of each fatigue test. The inset panel in A presents forces generated every second during the first 20 s of stimulation. The forces generated during the recovery phase were expressed as percentages of the peak force of the first tetanus recorded in the second test. CON refers to the non-supplemented group, CAR10W to the 10 weeks-supplemented group, and CAR34W to the 34 weeks-supplemented group.

During the first fatigue test, no group effect (F(2,1794) = 0.437, *p* = 0.647) nor group × time interaction (F(46,1794) = 0.794, *p* = 0.838; Figure 3A) were found. However, the force was lower during the first 20 s of the test in the CAR34W group than in the other two groups (effect of group F(2,1634) = 3.596, *p* = 0.032 and group × time interaction F(38,1634) = 3.195, *p* = 0.000; inset in Figure 3A). In the second fatigue test, when the lower (20 Hz) frequency of stimulation was applied, the level of forces was comparable in all groups (group effect, F(2,1817) = 0.215, *p* = 0.807; group × time interaction, F(46,1817) = 2.140, *p* = 0.063; Figure 3B). Finally, no statistical differences between groups were found during the recovery trains (group effect, F(2,462) = 1.216, *p* = 0.302; group × time interaction, F(12,462) = 0.522, *p* = 0.740; Figure 3C).

### 3.3. Antioxidant Potential, Advanced Glycation End Products, and Enzyme Activity

**Figure 4:**
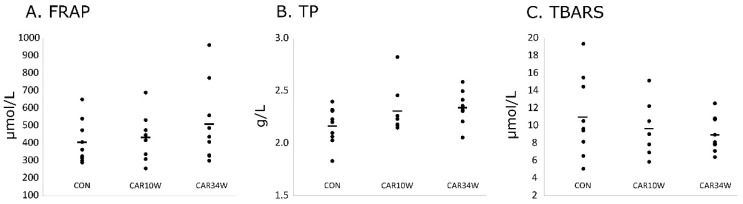
Comparison of the mean values of total antioxidant capacity, assessed with the ferric reducing ability of plasma (FRAP, **A**) method, as well as the total phenol substances (TP, **B**) and thiobarbituric acid reactive substances (TBARS, **C**) concentration in the medial gastrocnemius muscle between the studied groups. CON refers to the non-supplemented group, CAR10W to the 10 weeks supplemented group, and CAR34W to the 34 weeks supplemented group.

The total antioxidant capacity measured in the medial gastrocnemius muscle by FRAP did not significantly differ between the studied groups (F(2,23) = 0.887, *p* = 0.426, one-way ANOVA; Figure 4A). Similarly, there was no statistical difference between the mean values of the total phenol substances (TP) of all three groups (F(2,23) = 2.185, *p* = 0.135, one-way ANOVA; Figure 4B) and TBARS (F(2,22) = 0.788, *p* = 0.467, one-way ANOVA; Figure 4C).

There was no statistical difference in AGE content nor in the activity of kinase pyruvate or citrate synthase in MG muscle between the groups. However, the activity of hexokinase was lower in both experimental groups when compared to the CON group (*p* = 0.005 and *p* = 0.041 for CAR10W and CAR34W, respectively, post hoc Bonferroni-corrected *t*-test; Table 2).

### 3.4. Carnosine, Anserine, and Taurine Content in Muscle Samples

The carnosine content in the red portion of the medial gastrocnemius muscle decreased with supplementation (Table 3). Specifically, its content was significantly decreased by ~30% in the CAR34W group (post hoc Dunn’s test, *p* = 0.037), but not in the CAR10W group (post hoc Dunn’s test, *p* = 1.000) as compared to CON group. Further, the levels of anserine decreased with supplementation (Table 3), but, only when carnosine and anserine concentrations were pulled together, the overall level of histidyl dipeptides was found decreased in the CAR34W group (post hoc Bonferroni-corrected *t*-test, *p* = 0.0286), but not in the CAR10W group (post hoc Bonferroni-corrected *t*-test, *p* = 0.236) in comparison to the CON group. The concentrations of taurine were comparable in all three groups and amounted to about 20 mmol/kg WW (Table 3). 

In the white portion of MG muscle, we did not observe any changes in the levels of carnosine, anserine, and histidyl dipeptides. Similarly, the levels of taurine in both portions of MG muscle were similar in all three groups and equaled 33 mmol/kg WW (Table 3).

### 3.5. Body and MG Muscle Weight

**Figure 5:**
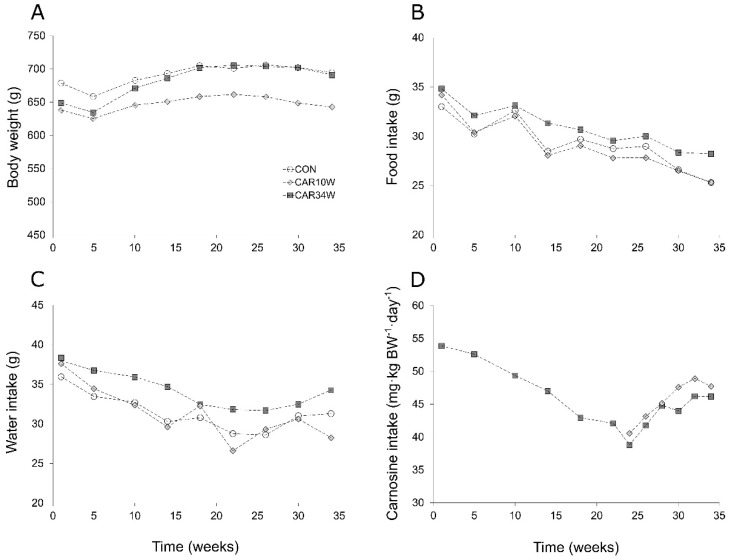
The mean values of body weight (**A**), as well as of food (**B**), water (**C**), and carnosine daily intake (**D**) in the three groups of animals, measured weekly throughout the whole study period. CON refers to the non-supplemented group, CAR10W to the 10 weeks supplemented group, and CAR34W to the 34 weeks supplemented group.

The mean body weight in animals that survived at the beginning and the end of the study was: 679 ± 69 g (SD) and 694 ± 90 g, 638 ± 84 g and 643 ± 53 g, and 649 ± 54 g and 691 ± 68 g in the CON, CAR10W, and CAR34W groups, respectively and it did not differ between groups during the whole study (repeated measures ANOVA, F(2,23) = 1.334, *p* = 0.283, Figure 5A). The mean weight of the studied MG muscle in the CON, CAR10W, and CAR34W groups was 0.988 ± 0.154, 0.703 ± 0.261, and 0.853 ± 0.140 g, respectively. The MG muscle weight in the CAR10W group was significantly lower than in the control group (one-way ANOVA, F(2,23) = 4.797, *p* = 0.0182, Figure 1A). However, when it was corrected for body weight with MG/body weight ratio, the values were not significantly different (one-way ANOVA, F(2,23) = 3.205, *p* = 0.0592, Figure 1B).

### 3.6. Food, Water, and Carnosine Consumption

Food consumption did not differ significantly between the groups during the course of the study (repeated measures ANOVA, F(66,396) = 0.699, *p* = 0.962; Figure 5B) and was, on average, 29.5, 29.1, and 31.2 g/day in the CON, CAR10W, and CAR34W groups, respectively. Similarly, water intake was not statistically different between the groups and amounted to 31.4, 31.7, and 33.8 g in the CON, CAR10W, and CAR34W groups, respectively (repeated measures ANOVA, F(66,462) = 0.788, *p* = 0.884; see Figure 5C). Finally, the mean values of carnosine intake were 47.2 mg/kg body wt/day in the CAR10W group and 47.9 mg/kg body wt/day in the CAR34W group and were not statistically different (*p* = 0.312, Mann–Whitney U test; Figure 5D).

### 3.7. Animal Behaviour and Mortality

We did not observe any abnormal signs in the appearance or the behavior of animals, such as body shape, body proportions, limb and fur appearance, and coloring, as well as the animals’ movements, response to handling, vocalization, grooming, eating or exploration.

The study was carried out for a relatively long time (34 weeks), which covered approximately 30% of the median Wistar rat lifespan [39]. In the CON group, 9 of 14 animals survived at the end of the study, which amounted to 64.3%. Similarly, in both experimental groups, the survival rate was 66.7% (8 of 12 rats) in the CAR10W group and 64.3% (9 of 14 animals) in the CAR34W group. Therefore, the mortality rate in our study was approximately 35% in all groups, which was below the expected 40% value in 23–24 month old male Wistar rats (Rj:WI, SPF Han; data obtained from Janvier Labs, France).

## 4. Discussion

This study has shown that carnosine supplementation induced very specific alterations in the fatigability of MUs. Specifically, in FF MUs, force was maintained at a higher level during the first 70 out of 240 s of initial fatiguing stimulation after both supplementation periods. Similar changes were not seen in other MUs. Chronic supplementation decreased activity of hexokinase, while it did not affect pyruvate kinase and citrate synthase activity. Short-term oral carnosine supplementation did not change, while long-term supplementation decreased muscle carnosine content, but only in the red portion of the studied muscle. Nevertheless, regardless of the time of supplementation, the survival rate, muscle antioxidant potential, and contractile properties of fast and slow MUs were not changed in aging rats.

We found some beneficial adjustments in fast MU fatigability in supplemented aged rats. This was especially seen in FF MUs after long-term supplementation and in FR units after short-term supplementation. It seems that the most pronounced improvements in MU contractility were observed in the fast units, which are composed of fatigable type II muscle fibers and are mostly located in a white part of MG muscle, where we did not observe any increase in histidyl dipeptides content. There was only one study in rodent model of aging (senescence-accelerated mice (SAMP8)) showing that histidyl dipeptides are involved in improving resistance to fatigue [40]; however, unlike in our study, muscle concentrations of histidyl dipeptides were elevated by creatine oral supplementation. Nevertheless, it was found in that study that this increase was also accompanied by the attenuation of relative forces between the 6th and 12th minute during repeated fatiguing stimulation of the soleus muscle. Importantly, these changes were observed only in short-term (15 weeks), and not in long-term (50 weeks), supplemented mice [40]. In our previous study in young rats, we also found that elevated muscle carnosine content through beta-alanine supplementation coincided with improved resistance to fatigue in both fast and slow MUs [18]. Our findings suggest that the beneficial effects of carnosine on muscle contractility are not necessarily accompanied with the detectible increase in muscle histidyl dipeptides concentrations, but possibly may be mediated through other mechanisms of action of these dipeptides. Furthermore, changes in muscle fatigability after carnosine treatment cannot also be attributed to positive antioxidant action of carnosine in the contracting muscle in old rats, which is not consistent with the data available in the literature for younger individuals [41].

In this study we observed that long-term supplementation of carnosine resulted in better force maintenance of FF MUs during the early stages of fatigue resistance, when phosphagens and glycolysis are the dominant energy source. Surprisingly, decreased activity of hexokinase, which is an enzyme necessary for phosphorylation of glucose during the first step of glycolysis [42], was observed. Hexokinase is a very important enzyme which determines the skeletal muscle ability to sustain contractile activity with moderate intensity [8]. Previous studies have suggested that the activity of hexokinase, as well as the two other enzymes measured in our study, decreases with aging [7]. We did not expect its lower values in supplemented animals in comparison to control rats. One could speculate that specific adaptations of many enzymes involved in muscle contraction occurred after carnosine treatment, as it was observed, for instance, after long-term strength training [43]. During the second fatigue protocol performed after 2 min of rest, carnosine supplementation resulted in lesser fatigability during the early period of muscle stimulation in FF MUs after 8 months of supplementation and in FR MUs after 10 weeks of supplementation. This effect was probably not due to better repletion of energy stores, as force started from the same level for both fast MU types after the 3 min rest break. It is possible that the MUs of supplemented rats may have become more insensitive to the fatiguing influences of phosphagen breakdown during repeated stimulation. The limitation of the current study is that we did not monitor specific indices in the blood of the animals which would be helpful in assessing the potential systemic effects of carnosine supplementation. 

Interestingly, twitch force was increased while force produced during the very initial stages of fatiguing stimulation protocol declined more in S MUs of rats supplemented for 8 months. This coincided with the decrease in the carnosine and histidyl dipeptides content in the red portion of medial gastrocnemius muscle. This finding suggests that changes in muscle carnosine content perhaps can affect excitation-contraction coupling of slow muscle fibers.

In the present study, we did not observe any differences with regard to antioxidant potential in the studied muscle (see Table 2 and Figure 4). Neither survival rates nor muscle weights were changed by long- and short-term carnosine supplementation. Carnosine has been broadly studied for in recent decades as a potential drug [44,45,46,47], as an important compound for muscle physiology [48], and also, to some extent, to establish if it can be used as treatment of age-related diseases [49,50]. However, there are still too few studies to definitively confirm or refute the potential of carnosine as an anti-aging drug. The study by Bingül et al. [51] performed on 20-month old rats has shown that a two-month carnosine treatment decreased reactive oxygen species formation in the serum and liver, as well as on AGE, malondialdehyde, protein carbonyl, and advanced oxidized protein product levels in aged rats. However, in this case, carnosine was administrated through intraperitoneal injection at 250 mg/kg body wt/day, which was five times more than in our study. Finally, we cannot exclude that antioxidant potential was increased in the blood. This can be effective for decreasing muscle fatigue, especially in subjects with low basal levels of antioxidants [52], which can be present during aging. However, unfortunately, in our study, we have not measured antioxidant potential in blood. Nevertheless, based on our data, it seems that oral carnosine supplementation is not able to increase life expectancy nor prevent muscle sarcopenia with aging.

To our knowledge, currently only a few studies have assessed carnosine content in adult rat skeletal muscles [53,54]. Nevertheless, it was generally shown that in rats there is a negative correlation between muscle carnosine concentration and aging [55], and that carnosine concentration in skeletal muscles is decreased in older individuals [40,56]. In our previous study, we found that the average values of histidyl dipeptides concentrations in the white gastrocnemius muscle of control young Wistar rats were 17 mmol/kg and 18 mmol/kg in wet muscle weight in red medial gastrocnemius muscle [18]. If we compare these results with the values measured in control aged animals from this study, we can note 25% lower HCD values in the white portion of MG muscle and even 50% lower values in the red MG in aged animals (13 and 9 mmol/kg wet wt, respectively). This observation is in line with the available data by Johnson and Hammer [56] and Derave et al. [40], who have found that the level of histidyl dipeptides declines in skeletal muscles by 35–50% with advancing age in rat and senescence-accelerated mice (SAMP8).

In this study, neither long-term nor short-term carnosine supplementation caused any increase in carnosine or anserine levels. Furthermore, decreased carnosine and histidyl dipeptides muscle content was found after long-term, but not short-term, supplementation (Table 3). These results suggest that, with a long period of carnosine supplementation, some compensatory mechanisms are initiated which regulate carnosine homeostasis. 

One explanation may be that after many months of carnosine supplementation, it was more energy-efficient not to increase, or even to decrease, histidyl dipeptides synthesis. There was probably also no need to maintain their higher levels in muscle tissue, as the accessibility of their precursors was higher than in the normal diet. Furthermore, even though there is no available data concerning the possibility of down-regulation mechanisms after chronic carnosine treatment, there are examples of such regulation in the case of other orally administrated drugs. Several studies suggested, for instance, that long-term creatine supplementation may result in insensitivity to creatine intake, which could probably be mediated through some tissue adaptations [57]. It was reported that chronic ingestion of creatine can down-regulate the expression of the creatine transporter [58] and, therefore, researchers suggest that the effects of long-term creatine supplementation on sarcopenia should be clarified [59]. It is possible that adaptation to chronic carnosine ingestion could also involve down-regulation of beta-alanine or histidine transporters. Saunders et al. [60] found that 24 weeks of beta-alanine supplementation in active males resulted in down-regulation of taurine transporter, which indicates that some alterations in gene expression may appear during long-term carnosine loading. In view of these and previous results showing the potential influence of carnosine on muscle physiology during aging [61], it seems to be important to establish the optimal dose and duration of carnosine treatment to obtain the most suitable biochemical and contractile adjustments in aged individuals.

The strength of the study was that the recordings of contractile activity were made separately for different phenotypes of MUs, innervating homogenous populations of muscle fibers and constituting the basic functional units responsible for generation of muscle force. The advantage of the applied experimental method was that most physiological processes were left intact. Therefore, this study evaluated in a more complex model the safety and neuromuscular efficacy of carnosine than in vitro studies or observational human studies. 

Although the concentration of dipeptides was assessed in muscle parts containing slow or fast muscle fibers, other biochemical measurements were made only for a whole muscle. The best approach would be to determine the studied effects of carnosine in muscle fiber samples belonging to specific MU phenotypes. Further, there was a relevant drop-out of studied subjects due to the mortality rate that approximated 35% and resulted in a decreased sample size. Although the results of our studies might be useful for better understanding carnosine action during aging, the direct extrapolation of our findings to humans cannot be made.

## 5. Conclusions

In conclusion, short- and long-term 0.1% oral carnosine supplementation in aged rats may be beneficial for partial counteraction of the rapid force decline of FF MUs, especially during functionally important, early stages (10–70 s) of fatiguing influences. Further, in FF, especially long-term, while in FR MUs, especially short-term, carnosine supplementation may have a positive effect on contractile function by attenuation of force decline after prolonged contractile activity. Based on our study, neither muscle antioxidant potential nor activity of specific glycolytic or oxidative enzymes can serve as mechanisms for the observed improvements in force generation during repeated contractile activity.

## Figures and Tables

**Table 1:** Contractile properties of sampled motor units of the left medial gastrocnemius muscle in CON (*n* = 6), CAR10W (*n* = 8), and CAR34W (*n* = 8) rats.

Motor Units Properties	Control (*n* = 94)	CAR10W (*n* = 95)	CAR34W (*n* = 97)	*p*
		FF		
TwF (mN)	72.3 ± 41.6	61.1 ± 39.6	74.0 ± 37.1	0.36
TetF (mN)	236.8 ± 138.3	214.3 ± 126.0	248.9 ± 125.4	0.52
CT (ms)	13.7 ± 1.8	13.0 ± 2.4	14.2 ± 2.9	0.18
HRT (ms)	20.4 ± 2.4	19.5 ± 2.4	20.9 ± 2.4	0.10
		FR		
TwF (mN)	32.7 ± 27.9	34.2 ± 21.2	40.0 ± 33.2	0.36
TetF (mN)	116.8 ± 87.2	144.8 ± 100.8	176.4 ± 163.6	0.25
CT (ms)	15.3 ± 3.2	15.9 ± 6.8	15.9 ± 4.1	0.61
HRT (ms)	21.5 ± 3.3	22.2 ± 6.0	22.2 ± 4.0	0.87
		S		
TwF (mN)	8.8 ± 3.4	11.4 ± 10.8	13.7 ± 6.3 *	0.00
TetF (mN)	63.3 ± 31.1	54.4 ± 44.6	66.1 ± 34.0	0.05
CT (ms)	25.6 ± 6.5	27.5 ± 7.5	27.1 ± 6.0	0.51
HRT (ms)	32.8 ± 6.8	34.5 ± 7.3	34.3 ± 5.2	0.54

Values are means ± SD. F, fast; FF, fast fatigable; FR, fast resistant; S, slow; TwF, twitch force; TetF, tetanus force; CT, contraction time; HRT, half-relaxation time. *p* refers to the one-way ANOVA or Kruskal–Wallis test for values where normality of data was not met or the variances were unequal. * *p* < 0.05 difference in relation to the control group, Dunn’s post hoc test. CON refers to the non-supplemented group, CAR10W to the 10 weeks supplemented group, and CAR34W to the 34 weeks supplemented group.

**Table 2:** Concentration of advanced glycation end products and the activity of hexokinase, kinase pyruvate, and citrate synthase in the left medial gastrocnemius.

	Control (*n* = 9)	CAR10W (*n* = 8)	CAR34W (*n* = 9)	*F*
AGEs (AU/mg of supernatant)	395.6 ± 28.90	386.2 ± 48.25	372.4 ± 31.36	0.91
Hexokinase (mU/mL)	9.19 ± 2.03	4.95 ± 3.22 ^#^	6.10 ± 2.04 ^#^	6.89 **
Pyruvate Kinase (mU/mL)	2.83 ± 1.02	3.01 ± 1.77	2.06 ± 1.15	1.25
Citrate synthase (U/mL)	30.1 ± 0.81	30.0 ± 1.35	29.0 ± 0.72	3.02

Mean values ± SD are presented. Calculated statistics: ** *p* < 0.001; *F*—one-way ANOVA test; ^#^ *p* < 0.05 refers to the difference in relation to the control group, Bonferroni-corrected *t*-test. A refers to Es-advanced glycation end products. CON refers to the non-supplemented group, CAR10W to the 10 weeks supplemented group, and CAR34W to the 34 weeks supplemented group.

**Table 3:** Muscle concentrations of carnosine, anserine, histidyl dipeptides (the sum of carnosine and anserine), and taurine (mmol/kg WW).

	Group	Red Gastrocnemius	Calculated Statistics	White Gastrocnemius	Calculated Statistics
Carnosine					
	CON	2.74 ± 0.46		2.13 ± 0.57	
	CAR10W	2.52 ± 1.00		2.15 ± 0.81	
	CAR34W	1.94 ± 0.45 ^#^	H = 6.34 *	2.04 ± 0.58	F = 0.06
Anserine					
	CON	6.20 ± 1.27		10.78 ± 2.20	
	CAR10W	5.04 ± 1.17		9.23 ± 3.21	
	CAR34W	4.96 ± 0.85	F = 3.70 *	8.60 ± 1.89	F = 2.00
Histidyl dipeptides					
	CON	8.94 ± 1.44		12.91 ± 2.69	
	CAR10W	7.57 ± 2.12		11.38 ± 3.99	
	CAR34W	6.90 ± 1.10 ^#^	F = 4.16 *	10.64 ± 2.18	F = 1.43
Taurine					
	CON	20.10 ± 4.41		32.65 ± 4.61	
	CAR10W	20.95 ± 6.23		33.29 ± 6.25	
	CAR34W	19.71 ± 3.04	F = 0.16	32.82 ± 2.90	F = 0.04

Mean values ± SD are presented. Calculated statistics: * *p* < 0.05; F—one-way ANOVA, H refers to the Kruskal–Wallis test for values where normality of data was not met or the variances were unequal. ^#^ *p* < 0.05, differences in relation to the control group, Bonferroni-corrected *t*-test, or Dunn’s post hoc test. CON refers to the non-supplemented group, CAR10W to the 10 weeks supplemented group, and CAR34W to the 34 weeks supplemented group.

## Data Availability

The datasets generated during and/or analysed during the current study are available from the corresponding author on reasonable request.
